# Muscle shortening in structural versus non-structural spine deformities

**DOI:** 10.1186/1748-7161-9-S1-O13

**Published:** 2014-12-04

**Authors:** Marianna Białek, Anna Brzęk, Ewelina Białek-Kucharska, Tomasz Kotwicki

**Affiliations:** 1 FITS Center, Jawor, Poland; 2 Institute of Kinesiology, Medical University of Silesia, Katowice, Poland; 3 Department of Pediatric Orthopaedics and Traumatology University of Medical Sciences, Poznań, Poland

**Keywords:** Spine deformities, idiopathic scoliosis, Scheuermann disease, length of the muscles.

## Background

Restriction of muscle flexibility is observed in lower limbs of both healthy adolescents and those diagnosed with spine deformity. Children sedentary lifestyle is suspected to be the reason. The resulting inhibition of antagonistic muscle group and disturbance of synergistic/stabilizing groups can potentially involve global muscular balance.

The aim of the study was to assess lower limb muscle shortening in children with spine disorders

## Methods

412 children, aged 10 to 16 years, mean = 12.6 ±1.8 were examined. The children were categorized into four groups: Group A – idiopathic scoliosis of 10 to 25 Cobb degrees, n=113, Group B – idiopathic scoliosis of more than 25 Cobb degrees, n=110, Group C – Scheuermann juvenile kyphosis, n=31, Group D – healthy control children age matched, n=158. One observer (first author) performed exam of all children using classical clinical tests to diagnose shortening over the following muscles: gastrocnemius, soleus, hamstrings, rectus femoris, adductor longus, adductor magnus, tensor fasciae latae and piriformis.

## Results

All children presented muscle shortening in lower limbs, most often in gastrocnemius, hamstrings and adductors (84.1% to 94.3% for all groups). Differences in muscle shortening prevalence were observed between the scoliosis groups and the Scheuermann group, p<0.001, paired t-test. No correlation was found between scoliosis angle and muscle shortening, Spearman 0.16, p>0.07. Piriformis revealed least shortening (22.6% to 34.5% of all subjects).

## Conclusions

1. Children without structural spine deformities presented limitation in all tested muscles.

2. Younger an adolescent, more often the muscles tested were short.

3. Every Scheuermann patient presented shortening in hamstrings, adductor longus and magnus.

4. Longitudinal data on lower limbs muscle shortening in adolescence is needed.

